# RGD-targeted redox responsive nano micelle: co-loading docetaxel and indocyanine green to treat the tumor

**DOI:** 10.1080/10717544.2021.1977425

**Published:** 2021-09-27

**Authors:** Lili Ren, Junfang Nie, Jie Wei, Yaning Li, Jun Yin, Xiaolong Yang, Guoguang Chen

**Affiliations:** a School of Pharmacy, Nanjing Tech University, Nanjing, China; b Department of Microbiology and Immunology, Stanford University, Stanford, CA, USA

**Keywords:** Polymer micelles, RGD peptide, redox responsive, combination therapy

## Abstract

Cancer, also known as a malignant tumor, has developed into a type of disease with the highest fatality rate, seriously threatening the lives and health of people. Chemotherapy is one of the most important methods for the treatment of cancer. However, chemotherapy drugs have some problems, such as low solubility and lack of targeting, which severely limit their clinical applications. To solve these problems, we designed a block copolymer that has a disulfide bond response. The polymer uses RGD peptide (arginine-glycine-aspartic acid) as the active targeting group, PEG (polyethylene glycol) as the hydrophilic end, and PCL (polycaprolactone) as the hydrophobic end. Then we utilized the amphiphilic polymer as a carrier to simultaneously deliver DOC (docetaxel) and ICG (indocyanine green), to realize the combined application of chemotherapy and photothermal therapy. The antitumor efficacy *in vivo* and histology analysis showed that the DOC/ICG-loaded micelle exhibited higher antitumor activity. The drug delivery system improved the solubility of DOC and the stability of ICG, realized NIR-guided photothermal therapy, and achieved an ideal therapeutic effect.

## 1. Introduction

At present, chemotherapy is one of the most commonly used treatments for cancer (Cornen & Vivier, 2018; Qin et al., 2018). However, some traditional chemotherapeutic drugs, such as paclitaxel (Heimans et al., 1994) and cisplatin (Jakse & Schmid, 1986), have poor water solubility and selectivity. As a result, these drugs have low bioavailability, cannot accumulate in tumor tissues, and are toxic to normal tissues (Yang et al., 2007; Zou et al., 2018; Dai et al., 2019). In addition, some molecular targeted therapeutic drugs that can selectively interfere with certain tumor-related specific receptors have been developed. But these molecularly targeted therapies are easily removed from the blood circulation in the body and cannot achieve the desired therapeutic effect (Lammers et al., 2008). Nanoparticle-based drug delivery systems (NDDS) can increase the water solubility of hydrophobic drugs, prolong blood circulation time and improve the permeability of drugs to tumors so that the drugs accumulate in the tumor, which is valued by researchers (Inoue et al., 2009; Su et al., 2016; Hu et al., 2017). In addition, nanometer photothermal adjuvants and chemotherapy drugs can also be delivered to the tumor at the same time. Under the irradiation of near-infrared light, photothermal adjuvants can convert light energy into heat energy, thus increasing the temperature of tumor tissue and killing tumor cells. Finally, the combinative antitumor effect of photothermal therapy and chemotherapy was realized (Luo et al., 2016; Yan et al., 2019).

An ideal nanodrug delivery system would selectively target tumor tissues (Gasparri et al., 2019; Jing et al., 2019; Piao et al., 2019). Compared with normal tissues, a solid tumor is rich in blood vessels, but the connections between these vascular endothelial cells are loose and the gap is larger. Therefore, some nanoparticles are easier to penetrate into the tumor tissue, which is the enhanced permeability and retention (EPR) effect of the tumor (Maeda et al., 2013; Yuxun et al., 2020). However, it is not enough to rely solely on the passive targeting of the tumor EPR effect, because some factors will limit the accumulation of nanoparticles under the EPR effect. These factors include early tumor metastasis is non-vascularized, some tumors have poor vascular permeability, and the pathophysiological characteristics of increased fluid pressure in the tumor interstitium (Zhao et al., 2018; Fang et al., 2020; Matsumura, 2020). Hence grafting on the surface of nanoparticles with active targeting ligands is a good solution. For example, arginine-glycine-aspartic acid (RGD) can bind to integrins on vascular endothelial cells. But it does not cross-react with platelet integrins and cellular receptors, so RGD-modified nanoparticles can target tumor angiogenesis (Song et al., 2016; Fang et al., 2019). Based on this, we grafted RGD onto the hydrophilic end of the polymer, enabling the polymer micelles to actively target tumor tissues.

Tumor tissues have unique microenvironments, such as low pH (Zhao et al., 2004; Huber et al., 2017), low oxygen (Dachset al., 1997; Patel & Sant, 2016; Huang et al., 2021), reductive properties (Yin et al., 2017), and over-expressed specific enzymes (Govindarajan et al., 2007; Li et al., 2018; Fujita et al., 2020), etc. In nanoparticle-based drug delivery systems, polymer micellar delivery strategies, which can design delivery carriers as amphiphilic polymer micelles that specifically respond to these stimuli, thereby achieving particularly drug release in the tumor environment (Cabral et al., 2018; Guo et al., 2018). The concentration of glutathione is significantly high in solid tumor tissues, resulting in a microenvironment with reducing properties. Therefore, we can design a disulfide bond in the di-block polymer. When the polymer micelle enters the tumor tissue, the disulfide bond breaks, so the drug in the micelle can be continuously released (Qu et al., 2016).

**Figure 1.**
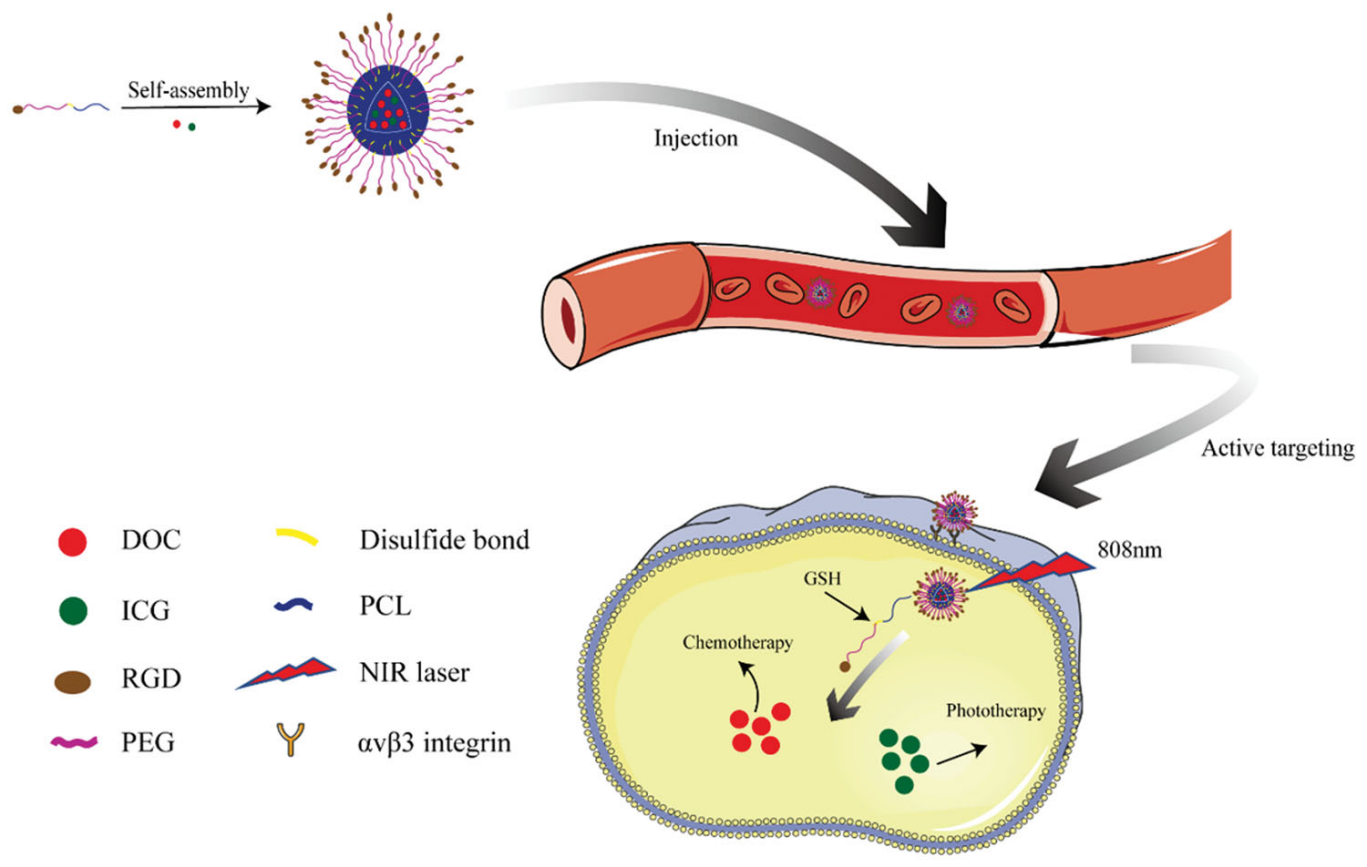
Illustration of RGD-PEG-ss-PCL micelles for targeted and triggered DOC/ICG delivery *in vivo*.

In this context, we designed and synthesized a drug nanocarrier PEG-ss-PCL with redox response, and grafted RGD peptide to the carboxyl modified PEG end. We characterized the blank micelles and studied the release of drug-loaded micelles under different conditions. After that, to prove the targeting effect of RGD and the effect of near-infrared light, we also carried out cell experiments and anti-tumor experiments *in vivo*. Figure 1 shows that the drug-loaded polymer micelles enter the tumor cells through passive targeting based on the EPR effect and the active targeting of RGD. Under the condition of the tumor microenvironment and near-infrared laser irradiation, the disulfide bond cleavage releases DOC and ICG to exert a therapeutic effect.

## 2. Experiment

### 2.1. Materials

Except for instructions, all reagents were purchased from Macleans. Glutathione (GSH), 3-(4,5-dimethylthiazol-2-yl)-2,5-diphenyltetrazolium bromide (MTT), fetal bovine serum (FBS), penicillin/streptomycin solution, RPMI 1640, phosphate-buffered saline (PBS), docetaxel (DOC), and indocyanine green (ICG) were purchased from Shanghai Yuanye Bio-Technology Co., Ltd. 4T1 cells were used as received. BALB/c mice were bought from Charles River.

### 2.2. Characterization of RGD-targeted amphiphilic polymer carrier (RGD-PEG-ss-PCL)

The synthesis of RGD-PEG-ss-PCL is provided in the supporting information. The composition of obtained RGD-PEG-ss-PCL (RPP) was affirmed by^1^H NMR spectra using an NMR spectrometer (Bruker AM300, Germany). IR spectra of the intermediates were confirmed by a Fourier-transform infrared spectrometer (Nicolet 5700, China).

The critical micelle concentration (CMC) of RPP was determined by UV spectrophotometry. The probe of this method is KI/I_2_ standard solution. When the absorbance of the polymer solution at 366 nm increases sharply, the concentration of the polymer corresponds to the CMC value of the micelle.

To verify the *in vivo* potential of RPP, we did a hemolysis assay using rabbit red blood cells. Mix equal volumes of RPP solution at different concentrations (0.5, 1, 2, 4, and 8 mg/mL) with 2% RBC solution. The mixture was incubated at 37° C for 1 h. And then, the supernatant was obtained by centrifugation. We using a UV–vis spectroscopy (Thermo, MK3, U.S.A.) to measure the absorbance of the supernatant at 540 nm. Here, the positive control is deionized water while PBS was regarded as a negative control. Hemolysis rates (HR) were calculated as follows:

$$HR\%=\frac{A_{\mathrm{sample}}-A_{\mathrm{negative}}}{A_{\mathrm{positive}}-A_{\mathrm{negative}}}\times 100\%$$

### 2.3. The preparation and characterization of blank micelle and drug-loaded micelle

The amphiphilic RGD-PEG-ss-PCL copolymer form micelles via self-assembly processes using the film hydration method in normal saline as described (Zhan et al., 2010). In typical procedures, RGD-PEG-ss-PCL copolymer (20 mg) was dissolved in 5 mL tetrahydrofuran (THF), spun, and steamed for an hour to form a film, and then add 5 mL saline. To prepare drug-loaded micelles, add DOC and ICG to the copolymer solution before steaming.

The morphologies of the micelles were acquired by transmission electron microscopy (JEM-2100). The size distribution and zeta potential of blank micelle and drug-loaded micelle were assessed by Laser Particle Analyzer (JL-1198).

### 2.4. Stability and reduction response of the micelle

To verify the stability of the RPP lyophilized powder when stored at 4 °C, the particle size changes of the RPP micelles were measured at 1, 3, 5, 10, and 15 days. DL-Dithiothreitol (DTT, 40 mM) was added to the 0.4 mg/mL of the micelle solution and then placed in a dark environment at 4 °C for 10 h. After the disulfide bond was fully broken, we will measure the change in the particle size of the micelle.

### 2.5. Loading and release of drug in micelles

We use the HPLC (LC-20A, Shimadzu, Japan) and UV–vis (UV-3200, China) to determine the drug loading (DL) and encapsulation efficiency (EE) of the drug-loaded micelles. The micelle is broken under ultrasound and vortex, thus the drugs in the micelles would be released. Then pass the 0.22 μm filter membrane to remove the insoluble matter. The mobile phase for the determination of DOC by HPLC is an acetonitrile/water (60:40, v/v) mixture and the detection wavelength is at 232 nm. The maximum absorption wavelength of ICG is 784 nm, so we use the absorbance of the solution at this wavelength to determine the concentration of ICG. The DL and EE values were valued as follows:

$$DL\%=\frac{\mathrm{amount}\;of\;\mathrm{drug}\;in\;\mathrm{micelles}}{\mathrm{amount}\;of\;\mathrm{micelles}}\times 100\%$$

$$EE\%=\frac{\mathrm{amount}\;of\;\mathrm{drug}\;in\;\mathrm{micelles}}{\mathrm{amount}\;of\;\mathrm{drug}\;\mathrm{feed}}\times 100\%$$

*In vitro* drug release from RPP micelles was investigated by using dialysis methods. 5 mL DOC and ICG loaded micelles (RPP@DOC/ICG) solution was placed in the medium of 50 ml for dialysis (MW 3500kD). The dialysate was a PBS solution of different pH (5.5, 6.5, and 7.4) and placed in a shaker (100 rpm, 37 °C for 72 h). We determined the amount of DOC and ICG released from the micelles by HPLC and UV–Vis, respectively. To study redox-responsive properties, RPP@DOC/ICG micelles were incubated in PBS with or without DTT (10 mM). To evaluate the effect of near-infrared light in drug release, RPP@DOC/ICG micelles were dispersed in PBS and irradiated by an 808 nm laser for 10 min.

### 2.6. Cellular uptake

The cells were seeded in glass-bottom petri dishes. When the cells adhered to the wall, they were further cultured in the medium containing DOC/ICG, PP@ DOC/ICG, and RPP@DOC/ICG. Fix cells with 4% paraformaldehyde and stain with Hoechst 33258 dye. The cellular uptake was observed by a CLSM (Olympus America, Melville, NY, USA).

### 2.7. *In vitro* cell cytotoxicity assay

Cytotoxicities of DOC/ICG, PP@ DOC/ICG, and RPP@DOC/ICG against 4T1 cells were evaluated by MTT assay. The cells were seeded in a 96-well culture plate with 10^4^ cells/well. After the cells adhered to the wall, we put the cells exposing to blank, DOC/ICG, PP@ DOC/ICG, and RPP@DOC/ICG solution with serial concentrations for 24 h, divided into light groups (808 nm, 800 mW/cm^2^, for 3 min) and non-light groups. At predetermined times, 20 μL of MTT solution (5 mg mL^−1^) was added and incubated for 4 h. Then we added 200 μL of DMSO to dissolve the metabolized product. Finally, the absorption of the metabolite at 630 nm was measured using a microplate reader (Thermo, MK3, USA).

### 2.8. *In vivo* anti-tumor assay

All animal procedures were performed by the Guidelines for Care and Use of Laboratory Animals of Nanjing Tech University and experiments were approved by the Animal Ethics Committee of Jiangsu Center for Safety Evaluation of Drugs. The BALB/c nude mice were housed in an environment with a temperature: 25 ± 2 °C, relative humidity of 50 ± 2%. The 4T1 tumor model was formed in the BALB/c nude mice. 0.1 mL 4T1 cell suspension (1 × 10^8^/mL) was inoculated into the left axilla of BALB/c nude mice.

When the tumor volume of the mice was larger than 100mm^3^, they were divided into six groups at random (*n* = 6). The six groups were injected with normal saline, free DOC/ICG, free DOC/ICG (with a laser), PP@DOC/ICG, RPP@DOC/ICG, and RPP@DOC/ICG (with laser) formulations through the tail vein, respectively. And the dose of DOC in the formulations is 10 mg/kg. At 1 h after injection, the group with laser were been irradiated by near-infrared laser (808 nm, 800 mW/cm^2^) for 10 min on the tumor site.

The first administration was recorded as day zero, and the drug was given every three days. The body weight and tumor volume of mice were measured before each administration. The mice were sacrificed on the 9th day.

### 2.9. Histology analysis

We performed histological analysis to assess the biosafety of the micelles. In detail, while the treatment assay *in vivo* was finished, the tumor tissue and the major organs were removed and soaked in 10% formalin, followed by dehydration and embedment in paraffin. The treated tumors and organs were cut into 5 μm slices. Use xylene to remove the paraffin in the slices, and then pass them through ethanol, finally immerse them in purified water. We performed a histopathological examination of the sections with hematoxylin-eosin (H&E) staining.

### 2.10. Statistics

Statistical tests were performed using the Student’s *t*-test, and the statistical significance for the differences group using SPSS software, and *p* < .05 was considered statistically significant.

## 3. Results and discussion

### 3.1. Characterization of RGD-PEG-ss-PCL

RGD-PEG-ss-PCL was synthesized through the reaction shown in Figure S1. We performed the following characterization of the synthesized intermediates and final products and studied the features of the polymer.

The FTIR spectra of COOH-PEG-COOH, Boc-NH-ss-PCL, and COOH-PEG-ss- PCL showed in Figure S2. In the spectrum of the three substances, the peak at 1125 cm^−1^ can be seen, which is characteristic of C–O–C in PEG. In the spectrum of Boc-NH-ss-PCL, the peak at 1541 cm^−1^ is a typical amide I characteristic peak. In the spectrum of COOH-PEG-ss-PCL, the peaks at 2945 and 2817 cm^−1^ prove the existence of PCL. In addition, the peak at 1724 cm^−1^ belongs to the C=O stretch of the carboxylic group on PCL. And the ^1^H NMR spectra proved the successful synthesis of the target product RGD-PEG-ss-PCL (Figure S3).

**Figure 2.**
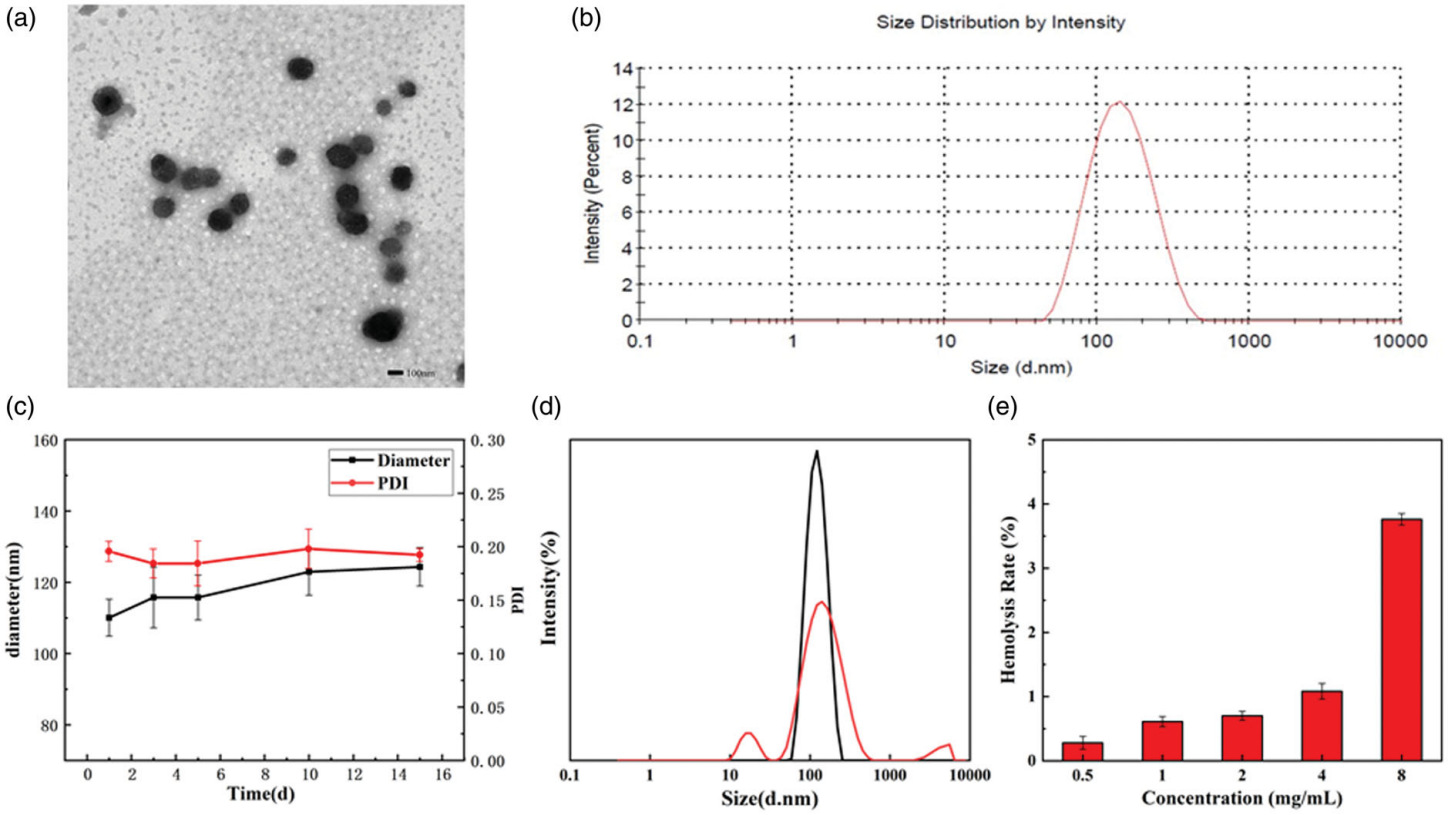
The TEM micrograph (a) and size distribution (b) of RPP@DOC/ICG micelles. (c) The change in particle diameter and PDI of RPP micelles. (d) The change of particle size distribution of blank micelles after DTT treatment. (e) Hemolysis rate of RPP. Data is represented as the mean ± standard deviation (n = 3)

**Figure 3.**
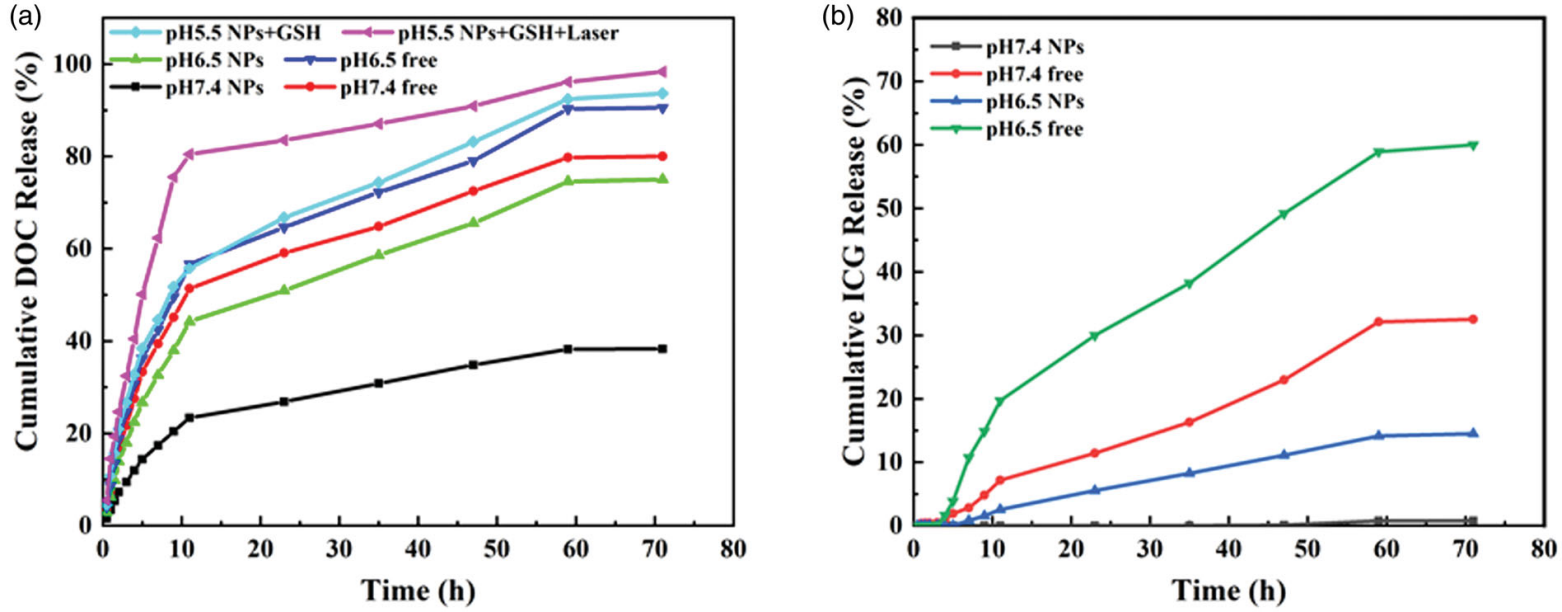
The release *in vitro* of DOC (a) and ICG (b) under different conditions.

### 3.2. Characterization of the micelles

TEM shows that the shape of RPP micelles is almost spherical (Figure 2(a)). The average particle size of RPP@DOC/ICG micelles measured by the Laser Particle Analyzer was 125.77 ± 8.18 nm, and the PDI was 0.193 (Figure 2(b)). Compared with RPP micelles (116.03 ± 6.56 nm, 0.186), the particle size of polymer micelles co-loaded with DOC and ICG was increased (Figure S4). We measured the zeta potentials of the drug-loaded micelles, where the potential of the DOC-loaded micelles is −2.05 mV and the potential of the DOC/ICG-loaded micelles is −1.58 mV (Figure S5). This is probably because ICG is encapsulated into micelles by electrostatic adsorption and the zeta potential is close to neutrality.

**Figure 4.**
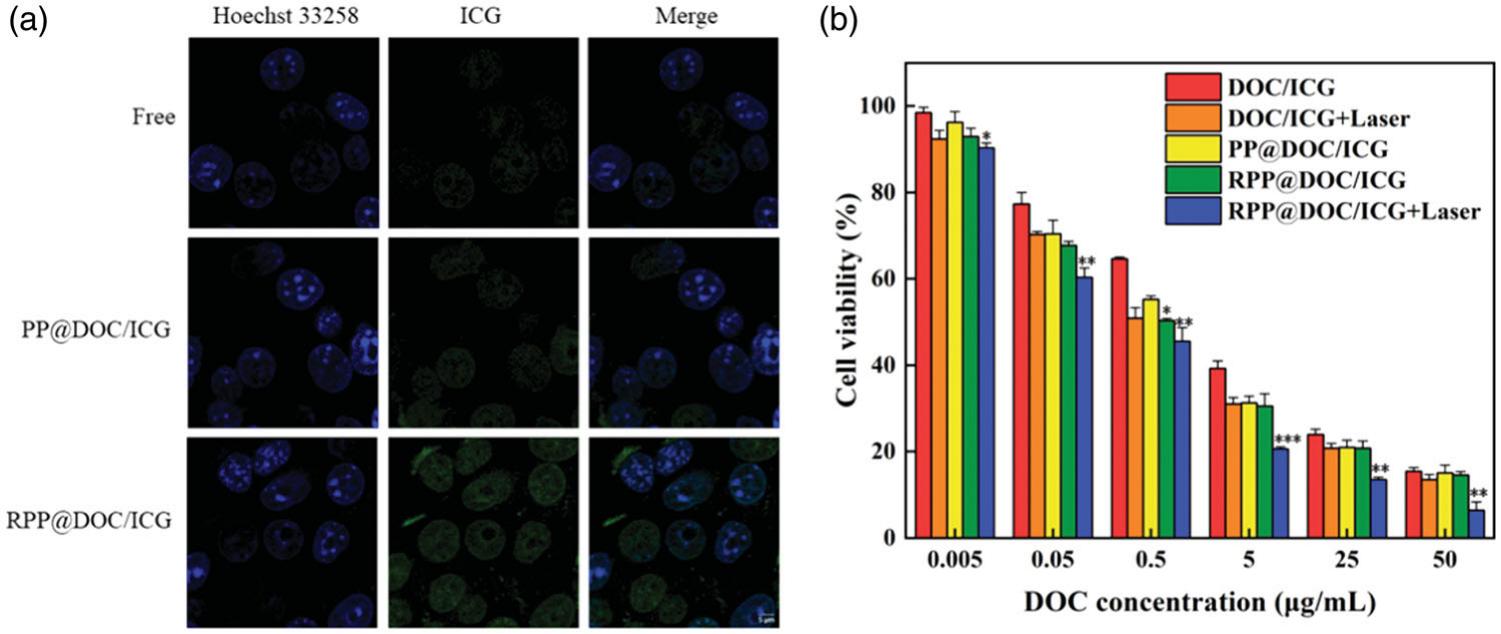
(a) Uptake of 4T1 cells for different formulations (Scale bar is 5 μm). (b) Cell viability of MTT assay. Data is represented as the mean ± standard deviation (*n* = 3), statistical significance compared with the PP@DOC/ICG group: *p < .05, **p < .01, ***p < .001.

**Figure 5.**
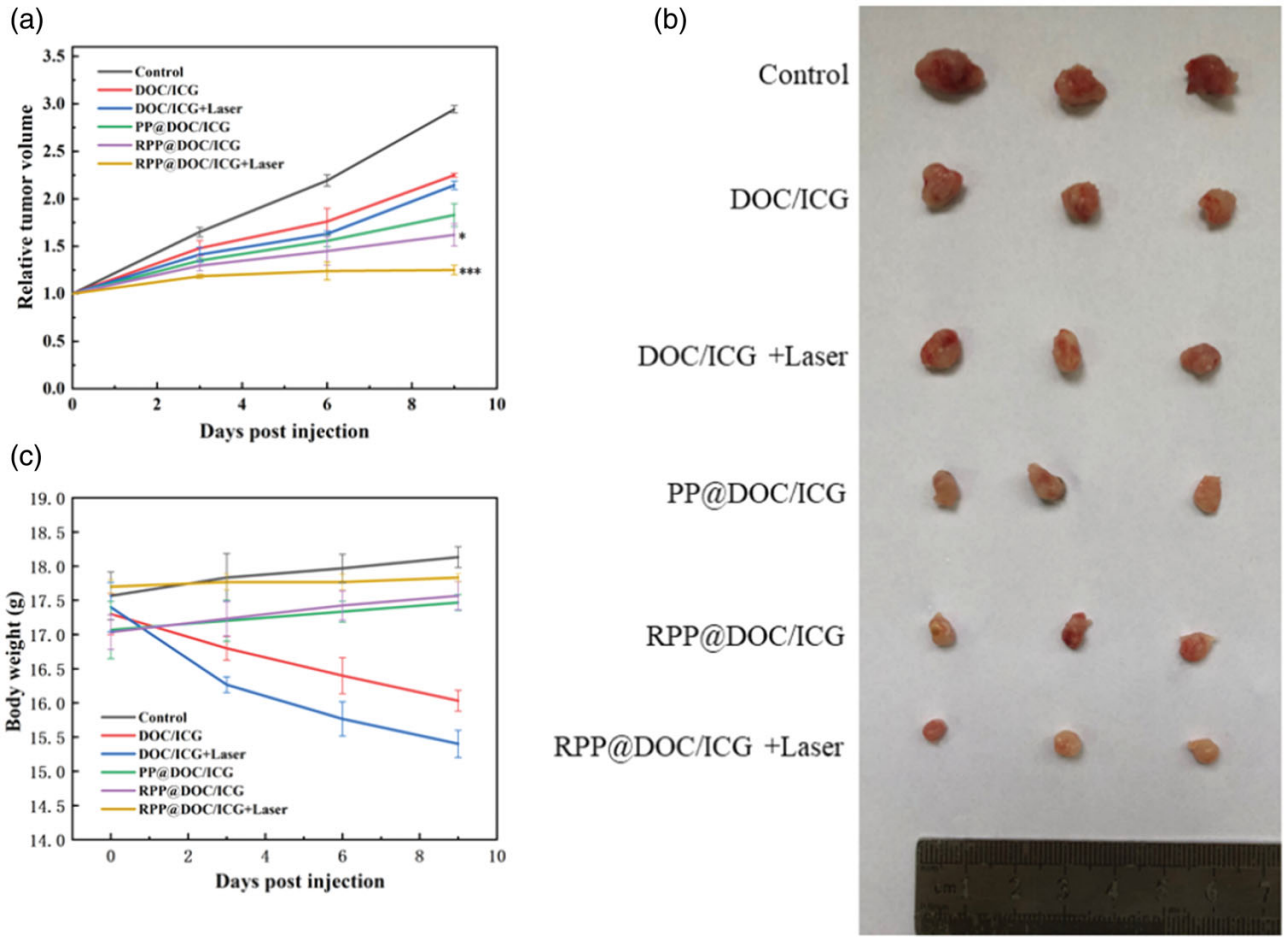
(a) The change of relative tumor volume. (b) Image of solid tumor. (c) The change of body weight. Data is represented as the mean ± standard deviation (*n* = 6), statistical significance compared with the PP@DOC/ICG group: *p < .05, ***p < .001.

The CMC value can reflect the stability of nano-micelles, and it was examined using UV spectrophotometry. The absorbance is positively correlated with the concentration of the polymer. When the polymer concentration rises to a certain value, the absorbance of the solution has an inflection point (Figure S6). The CMC value of RPP was about 0.35 mg mL^−1^, which is lower than PEG-(PCL)_8_ (0.40 mg mL^−1^) synthesized by Sytze Buwalda (Buwalda et al., 2018). The lower CMC makes it still stable even being diluted *in vivo*.

**Figure 6.**
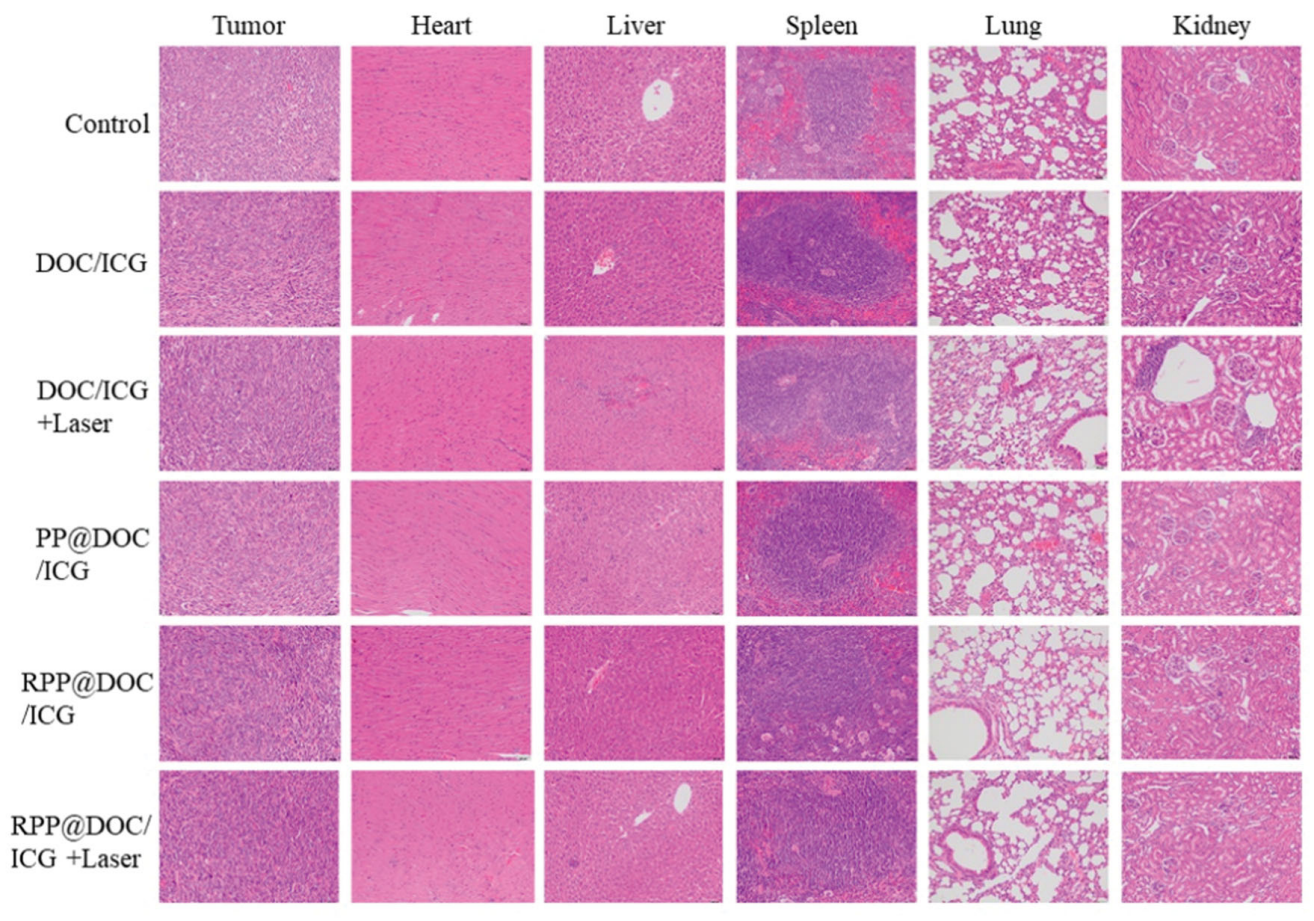
Tissue sections of tumor, heart, liver, spleen, lung, and kidney (magnification 200×).

We stored the micellar solution at 4 °C for 15 days and studied the change in particle size (Figure 2(c)). We can see that the size of polymer micelles increases slightly with the extension of storage time, and stabilize in the range of 100–125 nm. PDI does not change significantly within 15 days, and the micelles are stable. After the micelle solution is treated with DTT, it can be seen that there are particles with non-uniform particle size in the solution, and the original particle size distribution density has also decreased (Figure 2(d)). Therefore, it can be determined that the polymer micelles have reduced responsiveness.

### 3.3. Hemolysis assay

Before the pharmaceutical preparation is used for intravenous administration, a hemolysis test is generally required. This is to prevent the red blood cells from rupturing and causing hemolysis after the preparation is injected into the body. The hemolysis experiment of RPP is shown in Figure S7, and the calculated hemolysis rate is shown in Figure 2(e). When the concentration of RPP is 8 mg/mL, the hemolysis rate of the sample is 3.76% (<5%). So we can conclude that when the concentration of RPP is <8 mg/mL, it can be used for intravenous administration.

### 3.4. Loading and release

We measured the drug loading (DL) and encapsulation efficiency (EE) of DOC to evaluate the drug loading performance of micelles. And DL and EE of DOC were 9.87 ± 1.07 and 89.99 ± 2.08%, respectively. This means RPP will be apt to deliver DOC and ICG.

The release *in vitro* of DOC and ICG from RPP@DOC/ICG micelles was studied at 37 °C in PBS buffer under different conditions. As we can see in Figure 3(a), after 72 h in the medium of pH 7.4, the cumulative release rate of DOC in the DOC/ICG was as high as 80.0%. In contrast, the cumulative release rate of DOC from RPP@DOC/ICG micelles was only 38.3% under the same conditions. From this, we can easily infer that the polymer carrier can better prevent drug leakage in blood circulation. After 72 h in the medium of pH 6.5, the cumulative release rates of DOC in RPP@DOC/ICG micelles and DOC/ICG were 75.0 and 90.5%, respectively. The release rate of DOC was accelerated, which indicated that acidic pH was beneficial to the release of DOC. The mechanism that DOC can be better released under acidic conditions is not clear. In addition, the effect of GSH and near-infrared light (808 nm, 0.8 W cm^−2^) on drug release was evaluated. We can get that in the presence of GSH and without infrared light irradiation, after 12 h, the cumulative release rate of DOC is 55.8%, and at 72 h the value is 93.6%. While infrared irradiation, the cumulative release rate of DOC reached 80.5% at 12 h and 98.3% at 72 h. We can attribute this to the fact that the photothermal effect of ICG is facilitated drug release of drug-loaded micelles under infrared irradiation (Zhan et al., 2020).

The half-life of free indocyanine green *in vivo* is only 2–4 min (Sheng et al., 2013). The ideal carrier can not only efficiently encapsulate ICG but also stay stable in the process of blood circulation to avoid premature exposure to ICG. The release behavior of ICG in RPP micelles was determined by *in vitro* release assay, and the retention ability of RPP materials to ICG was evaluated (Figure 3(b)). In the medium of PBS (pH 7.4), the cumulative release rate of ICG after 72 h in RPP micelle was only 0.8%, while the cumulative release rate of DOC/ICG was as high as 32.5%. At pH 6.5, the cumulative release rate of ICG after 72 h in RPP micelle was 14.5%, and the cumulative release rate of ICG in the DOC/ICG group was as high as 60.0%. Therefore, under physiological conditions, polymer micelles can effectively retain ICG to avoid leakage in the process of circulation *in vivo*.

*In vitro* release assay confirmed that RPP@DOC/ICG micelles responses to GSH and the release of DOC is better under acidic conditions. It also can effectively avoid being cleared in the process of the systemic circulation, which would ensure a therapeutic effect on the tumor.

### 3.5. Cellular uptake

We used 4T1 cells as the object and studied the cell uptake behavior of DOC/ICG, PP@DOC/ICG micelles, and RPP@DOC/ICG micelles (Figure 4(a)). The cells in the RPP@DOC/ICG group had stronger green fluorescence, indicating that the drug-loaded micelles were endocytosed by 4T1 cells. However, there is only a small amount of green fluorescence inside the cells in the DOC/ICG group and PP@DOC/ICG group, which shows the drug is rarely taken up by cells. The high expression of αvβ3 integrin on the surface of 4T1 cells has been confirmed by many researchers (Mi et al., 2006; Jahanban-Esfahlan et al., 2017; Kebebe et al., 2019; Gao et al., 2020). The high uptake behavior in the RPP@DOC/ICG group may be attributed to RGD targeting.

### 3.6. MTT assay

The *in vitro* cytotoxicity of RPP@DOC/ICG micelles against 4T1 cells was evaluated by MTT assay. In various preparations, the viability of 4T1 cells decreased with the increase of drug concentration (Figure 4(b)), and the IC50 values are summarized in Table 1. We can see that the IC50 value of DOC/ICG with laser is obviously lower than that of DOC/ICG, which indicates that after ICG is given, infrared light can effectively inhibit the growth of tumor cells. For the PP@DOC/ICG, the IC50 value of the RPP@DOC/ICG decreased, indicating that the embedding of the targeting peptide RGD is beneficial to the uptake of drugs. Therefore, it is more effective to prevent the growth of tumor cells. Compared with the other groups, the IC50 value of the RPP@DOC/ICG with laser group was <0.3 μg/mL, which was significantly lower than the other groups. Therefore, we can infer that after the polymer carrier grafted with the targeting peptide RGD and given infrared light, the cytotoxicity of DOC to 4T1 cells was enhanced.

**Table 1.** IC50 values of different DOC formulations against 4T1 cells.

Formulation	IC_50_ (ug/mL)
DOC/ICG	1.73 ± 0.26
DOC/ICG + Laser	0.67 ± 0.04***^1^
PP@DOC/ICG	0.87 ± 0.04
RPP@DOC/ICG	0.64 ± 0.07*^2^
RPP@DOC/ICG + Laser	0.27 ± 0.06***^2^

Data is represented as the mean ± standard deviation (*n* = 3). Statistical significance ‘^1^’ is compared with the DOC/ICG group, and ‘^2^’ is compared with the PP@DOC/ICG group: *p < .05, ***p < .001.

### 3.7. *In vivo* antitumor assay

As the MTT assay showed the inhibitory effect of the micelles against 4T1 cells is effective, the antitumor behavior of the micelles *in vivo* was evaluated. The anti-tumor effect was evaluated by subcutaneous xenograft 4T1 tumor model in nude mice. It can be seen from the relative tumor volume-time curve that compared with the groups containing DOC/ICG, the tumors in the control group grew rapidly (Figure 5(a)). It proves that different micelles and DOC/ICG can inhibit the growth of tumors to different degrees. And the relative tumor volume of the groups of different micelles is smaller than the group of free drugs. Among them, the tumor suppression rate in the RPP@DOC/ICG with laser group reached 57.91 ± 1.88%, which was higher than that in the DOC/ICG with laser group (27.83 ± 2.13%) (Table 2). We can conclude that while the micelles co-loading DOC and ICG, the anti-tumor effect is better under the irradiation of laser. At the same time, the relative tumor volume of RGD-mediated drug-loaded micelles was significantly lower than non-RGD-mediated drug-loaded micelles. This is probably because RGD-modified drug-loaded micelles can recognize and bind to receptors on tumor cell membranes to improve transport efficiency.

**Table 2.** Average tumor inhibition rate.

Group	DOC/ICG	DOC/ICG + Laser	PP@DOC/ ICG	RPP@DOC/ ICG	RPP@DOC/ ICG + Laser
Tumor inhibition rate (%)	24.04 ± 1.41	27.83 ± 2.13	39.12 ± 1.95	44.02 ± 3.00	57.91 ± 1.88

Data is represented as the mean ± standard deviation (*n* = 6).

Figure 5(b) is the picture of tumors from different groups. From the weight of the mice, we can find that the weight of mice in free DOC/ICG has decreased markedly, while the weight of mice in the normal saline group and groups of the micelles has kept increasing slowly (Figure 5(c)). Compared with the free drugs group, the drug-loaded micelles showed low toxicity, which demonstrated that the polymer material showed the advantage of reducing the toxicity of the drug system and might act as a delivery system with a good prospect in the application.

### 3.8. Histology analysis

The biosafety of the micelles was evaluated by H&E staining analysis (Figure 6). After being treated with DOC/ICG and DOC/ICG-loaded micelles, the histological characteristics of the tumor were significantly different from those of the negative control group. The tumor cells shrank, the nucleus was condensed and even fragmented, indicating that the cells were apoptotic. As H&E staining analysis shows, in the free drug group, liver and kidney inflammation can be observed, indicating DOC has a certain degree of damage to the liver and kidney. However, there is no obvious abnormality or lesion in the main organs of the micelle group, which indicates that the organ toxicity of PP@DOC/ICG and RPP@DOC/ICG micelles is negligible.

## 4. Conclusion

In summary, we have successfully prepared RGD-targeted amphiphilic polymer materials, which can successfully form nano micelles to deliver DOC and ICG *in vivo*. We demonstrated that this micelle can increase the solubility of the drugs, improve tumor targeting, and reduce system toxicity. It also significantly improves the stability of ICG and achieves the combined anti-tumor effect of chemotherapy and photothermal therapy. The results show that the redox polymer micelles designed by us have the potential to be used as carriers for tumor-specific delivery and release of anticancer drugs.

## Supplementary Material

Supplemental Material
