# Supplementary material for: RGD-targeted redox responsive nano micelle: co-loading docetaxel and indocyanine green to treat the tumor
Source: Drug Deliv. 2021 Sep 27;28(1):2024–32. doi: 10.1080/10717544.2021.1977425 (PMC8477929; doi:10.1080/10717544.2021.1977425)
Supplement: Supplemental Material [file IDRD_A_1977425_SM2811.docx]

**RGD-targeted redox responsive nano micelle：Co-loading Docetaxel and Indocyanine Green to treat the tumor**

SUPPORTING INFORMATION

Lili Ren ^1,2^,Junfang Nie ^1^ , Jie Wei^1^ ,Yaning Li^1^，Jun Yin ^1^，Xiaolong Yang ^1^ and Guoguang Chen^1*^

1 School of pharmacy, Nanjing Tech University, 5th Mofan Road, Nanjing, 21009, China

2 Department of Microbiology and Immunology, Stanford University, Stanford, CA 94305, USA

* Correspondence ggchen@njtech.edu.cn (G,Chen); Tel.: +86-2558-139-416

**The synthetic route**

**The synthesis of COOH-PEG-COOH.**

Dissolve PEG and succinic anhydride in DCM, stir and reflux at 45°C for 48 hours under a nitrogen environment, and then add 20 mL of distilled water, continue to stir for 10 minutes, and separate the organic floor. After washing, drying, filtering, concentrating the filtrate, precipitating in ether and vacuum drying to obtain a white powder, which is COOH-PEG-COOH.

**The synthesis of Boc-NH-PEG-NH_2._**

Cystamine dihydrochloride was dissolved in acetone, triethylamine was added and stirred for 10 minutes, and then the di-tert-butyl bicarbonate dissolved in methanol solution was slowly dripped into the reaction solution. After stirring at room temperature for 3 hours, the reaction solution was concentrated. 1M NaH_2_PO_4_ was added, and then washed the solution with n-hexane / ethyl acetate (1:1,v:v), and the Boc-NH-PEG-NH_2_ was extracted with ethyl acetate.

**The synthesis of Boc-NH-ss-PCL.**

Boc-NH-ss-PCL was synthesized by ring-opening polymerization of ε-caprolactone using Boc-NH-ss-NH_2_ as substrate and SnCl_2_ as catalyst. The process is as follows: Boc-NH-ss-NH_2_, ε-caprolactone and SnCl_2_ were added to the eggplant-shaped bottle, dried at room temperature for 5 h, then reacted at 140 ℃ for 6 h, dissolved with dichloromethane, and dropped into excess ice ethanol to precipitate the product.

**The synthesis of COOH-PEG-ss-PCL.**

Dissolve Boc-NH-ss-PCL with THF and add 1M hydrochloric acid solution. The reaction was carried out at the room temperature in nitrogen for 12 h, and the solvent was removed by rotary steaming. Dichloromethane, triethylamine, COOH-PEG-COOH, 4-dimethylaminopyridine and dicyclohexyl -carbodiimide were added respectively, and the reaction continued for 48 h at room temperature. Add distilled water to stop the reaction. After filtration, the filtrate was decompressed and concentrated, then dissolved with ethyl acetate, placed at 4 ℃ for 12 hours, the precipitated solid was removed, concentrated the filtrate, the filtrate was re-dissolved with dichloromethane, precipitated with a large amount of ice ethanol and filtered. The product COOH-PEG-ss-PCL was obtained by repeatedly dissolving and precipitating for three times, drying under reduced pressure and overnight.

**The synthesis of RGD-PEG-ss-PCL.**

COOH-PEG-ss-PCL, EDC, and NHS (1:3:1.5, molar radio) were dissolved in DCM. After activation for 2 h, RGD was added, and the pH was adjusted to between 7.4-8 with triethylamine, and the reaction was carried out at room temperature for 24 h. Finally, the reaction solution was dialyzed (MWCO 3.5 kDa) for 24 hours and lyophilized to obtain the target product.





Figure S1. The Synthetic of polymer material (RGD-PEG-ss-PCL).


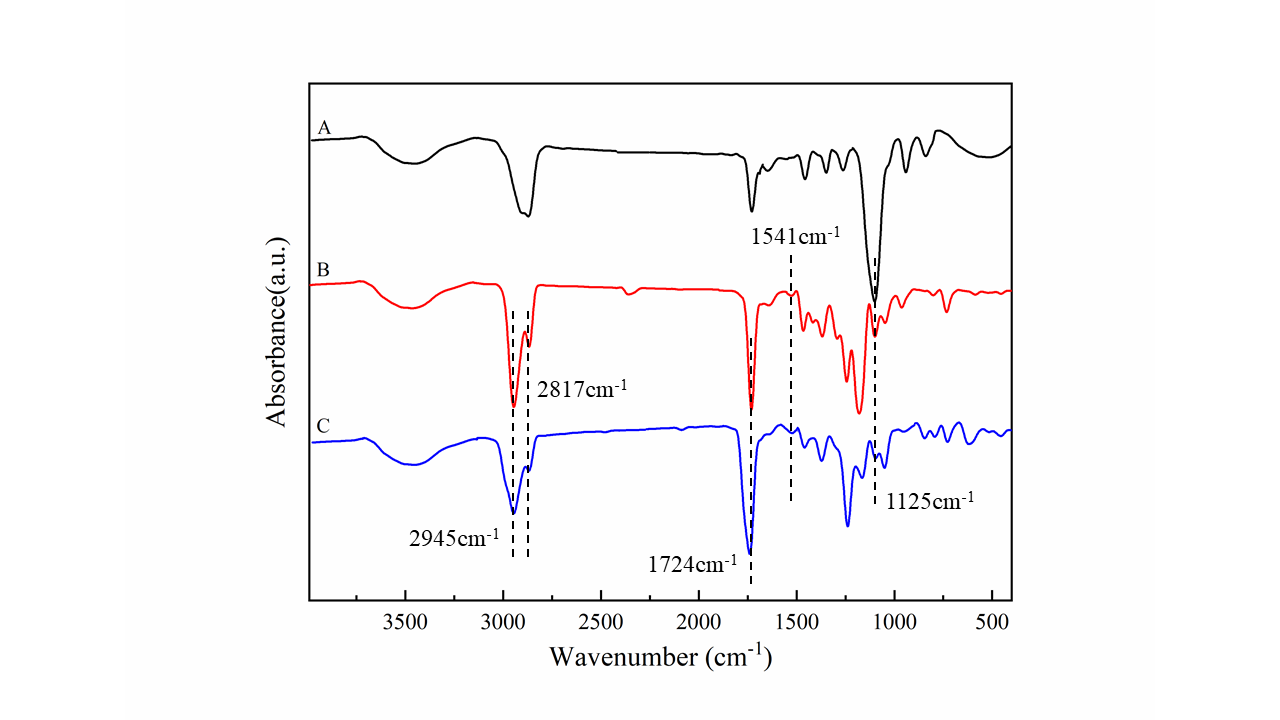


Figure S2. FT-IR spectra of COOH-PEG-COOH (A), Boc-NH-ss-PCL (B) and COOH-PEG-ss-PCL (C).


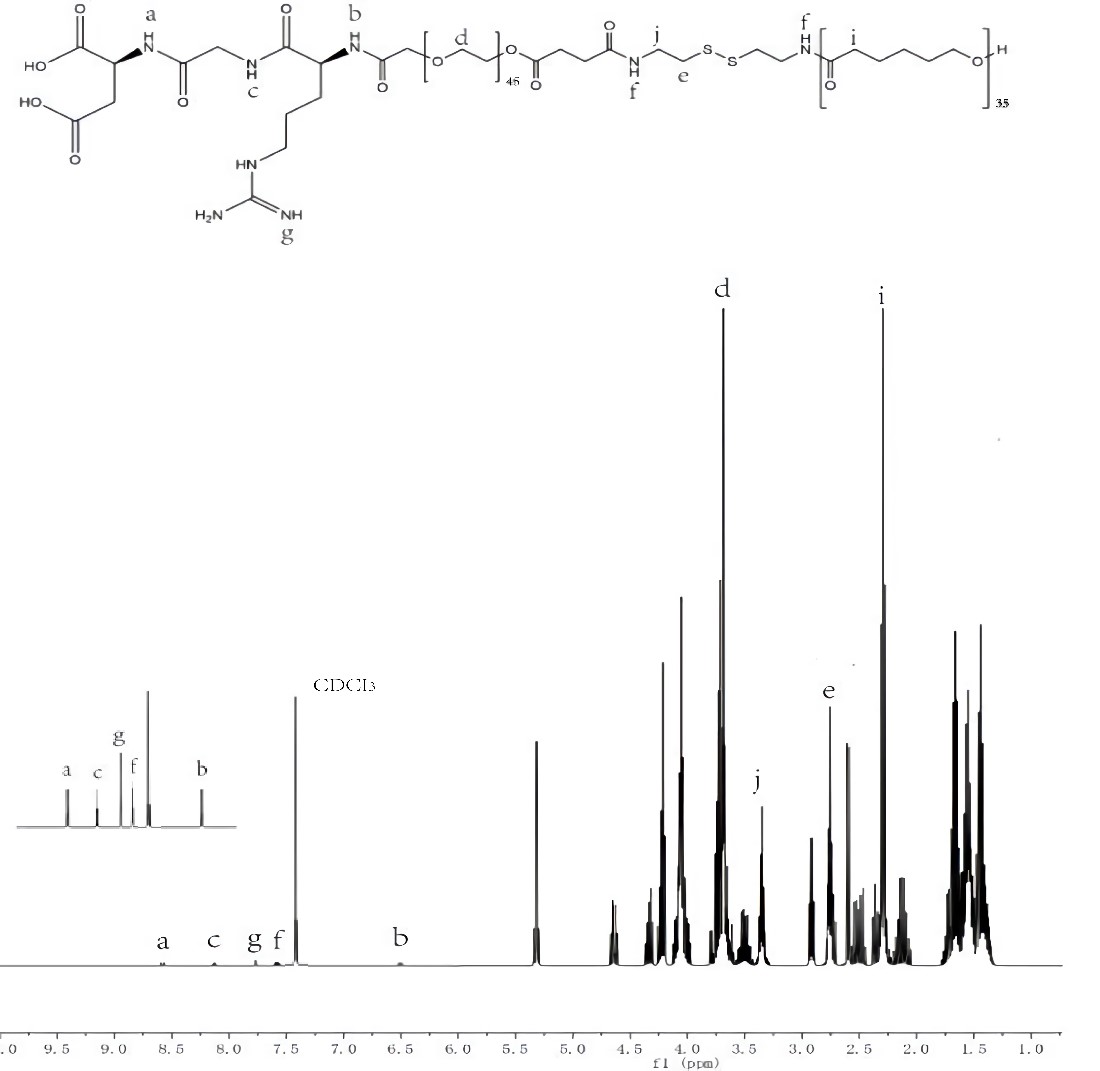


Figure S3. ^1^H NMR spectrum of RGD-PEG-ss-PCL.

The appearance of δ 9.41 (a, -NHCO-), δ 6.50 (b, -NHCO-), δ 8.17 (c, -NHCO-) and δ 7.76 (g, -C=NH) prove the successful connection of RGD and COOH-PEG-ss-PCL. The hydrogen proton shift at δ 3.65(d, -OCH_2_CH_2_-) indicates the existence of PEG, while δ 2.30 (i, -COCH_2_CH_2_CH_2_CH_2_CH_2_O-) indicates the existence of PCL. δ 7.61 (f, -NH-), δ 3.35 (j, -CH2-), δ2.66 (e, -CH_2_-ss-) are the hydrogen proton peaks near the disulfide bond.


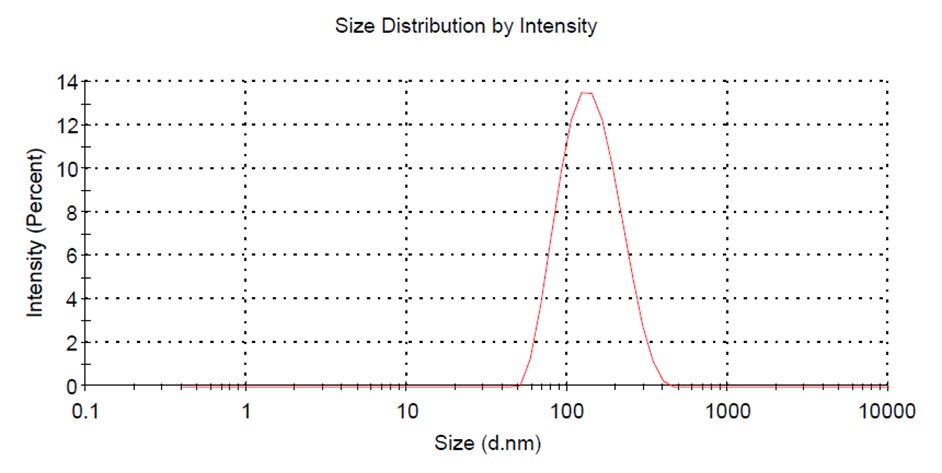


Figure S4. Size distribution of RPP micelles.





Figure S5. Zeta potential of RPP@DOC, RPP@DOC/ICG.





Figure S6. The relationship between absorbance and concentration of polymer.


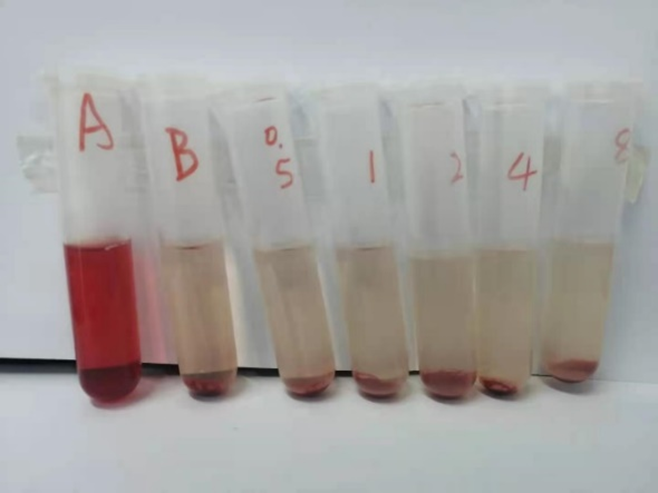


Figure S7. Hemolysis test (A: water, B: normal saline, and the others is the experimental group, which the concentration of RPP is 0.5, 1, 2, 4, 8 mg/mL).
